# Distinct Modulation of High- and Low-Frequency Activity by Midazolam and Neurosteroids in Neocortical Slices

**DOI:** 10.3390/ijms27156941

**Published:** 2026-08-02

**Authors:** Stefan Bieletzki, Clara Gotthard, Xènia Puig-Bosch, Hanns Ulrich Zeilhofer, Uwe Rudolph, Gerhard Rammes, Bernd Antkowiak, Berthold Drexler

**Affiliations:** 1 Department of Anesthesiology and Intensive Care Medicine, Experimental Anesthesiology Section, Eberhard Karls University, 72076 Tübingen, Germany; stefan.bieletzki@t-online.de (S.B.); clara.gotthard@med.uni-tuebingen.de (C.G.); bernd.antkowiak@uni-tuebingen.de (B.A.); 2 Department of Anesthesiology and Intensive Care Medicine, Medical School, Klinikum rechts der Isar, Technical University Munich, 81675 Munich, Germany; xenia.puig@ub.edu (X.P.-B.); g.rammes@tum.de (G.R.); 3 Artificial Intelligence in Medicine Lab (BCN-AIM), Departament de Matemàtiques i Informàtica, Universitat de Barcelona, 08007 Barcelona, Spain; 4 Institute of Pharmacology and Toxicology, University of Zurich, 8057 Zurich, Switzerland; zeilhofer@pharma.uzh.ch; 5 Institute of Pharmaceutical Sciences, Swiss Federal Institute of Technology (ETH) Zürich, 8092 Zürich, Switzerland; 6 Department of Comparative Biosciences, College of Veterinary Medicine, and Carl R. Woese Institute for Genomic Biology, University of Illinois at Urbana-Champaign, Urbana, IL 61801, USA; urudolph@illinois.edu

**Keywords:** neocortex, sedation, electroencephalographic activity, GABA_A_-subtypes, translocator protein (18 kD), up-states, organotypic cultures

## Abstract

Adverse effects of benzodiazepines are largely mediated by GABA_A_ receptors containing α1-subunits. Recent efforts to replace benzodiazepines with neurosteroids are promising, but it is unclear to what extent α1-GABA_A_ receptors are also upregulated by neurosteroids. Here, we investigate how a prototypical benzodiazepine and neurosteroids modify a form of neuronal activity that is controlled by α1-GABA_A_ receptors. We compared the actions of the benzodiazepine midazolam, the naturally occurring neurosteroid allopregnanolone, and XBD173, a drug that enhances neurosteroidogenesis, on high- (HFA, >50 Hz) and low- (LFA, <50 Hz) frequency action potential activity in cultured neocortical tissue slices derived from wild-type mice via extracellular multi-unit recordings. The effects of midazolam were also quantified in slices from α2/3/5-knock-in-mice, in which the drug acts almost exclusively via α1-GABA_A_ receptors. In slices derived from α2/3/5-knock-in mice, low nanomolar concentrations of midazolam suppressed HFA in a selective manner. In slices derived from wild-type animals, HFA was also attenuated by midazolam. In contrast, allopregnanolone and XBD173 did not affect HFA but strongly suppressed LFA. Our findings suggest that, in contrast to benzodiazepines, neurosteroids do not dampen HFA, a type of cortical network activity tightly controlled by α1-GABA_A_ receptors.

## 1. Introduction

The World Health Organisation classifies benzodiazepines as essential drugs for the treatment of anxiety, seizures and muscle tension. Furthermore, benzodiazepines are frequently used for sedating patients in and outside the operating room. They act via positive allosteric modulation of GABA_A_ receptors [1]. These receptors are heteropentamers composed of 19 known subunit proteins [2]. Their assembly from different subunit proteins results in different receptor subtypes displaying cell-type- and tissue-specific expression patterns [3]. Benzodiazepine-sensitive GABA_A_ receptors have two α-subunits (α1, α2, α3, or α5), two β-subunits, and a γ-subunit. The most common benzodiazepine-sensitive receptor subtype found in the forebrain contains an α1-subunit (α1-GABA_A_ receptors) and contributes to the drugs’ sedative and amnestic properties [1].

It is important to mention that adverse effects, including addiction [4] and tolerance development [5], are also linked to α1-GABA_A_ receptors, thereby limiting the therapeutic benefits of benzodiazepines. Therefore, the search for substances that have a similar effect profile to benzodiazepines, but spare α1-GABA_A_ receptors, appears to be promising. Furthermore, it has been proposed that in the future, some benzodiazepines could be replaced by neurosteroids with fewer side effects [6]. Similar to benzodiazepines, neurosteroids act as positive modulators at GABA_A_ receptors and seem to display little selectivity across different receptor subtypes [7]. However, unlike benzodiazepines, neurosteroids are effective at extrasynaptic GABA_A_ receptors containing δ-subunits [8], but it is unknown whether α1-GABA_A_ receptors are also among their preferred molecular targets.

Sedation caused by benzodiazepines is accompanied by prominent changes in brain activity [9]. In particular, the high-frequency neuronal population activity in the cerebral cortex, a well-known signature of conscious cognitive functions, appears to be downregulated. To investigate how benzodiazepines shape the activity patterns of cortical neurons via α1-GABA_A_ receptors, we previously analysed the effects of diazepam in a mouse model in which the drug exclusively acts via α1-GABA_A_ receptors [10]. In this study, micro-electrodes were chronically implanted in the mouse somatosensory cortex. When recording local field potentials, a correlate of synaptic population activity, diazepam diminished power spectral density in the γ-range (20–90 Hertz). Furthermore, the drug attenuated spontaneous multi-unit action potential firing.

Additionally, we explored how action potential activity is modulated by zolpidem, a hypnotic drug that acts mainly via α1-GABA_A_ receptors, in tissue slices from the mouse neocortex [11]. In this study, high-frequency action potential activity (HFA), but not low-frequency action potential firing (LFA), was strongly depressed by zolpidem. The conclusion that this effect was mediated by α1-GABA_A_ receptors was confirmed via experiments conducted on tissue slices derived from α1(H101R)-knock-in mice, in which α1-GABA_A_ receptors were present but resistant to zolpidem.

In the present study, using HFA as a biomarker for drug effects mediated via α1-GABA_A_ receptors, we compare the effects of (1) midazolam, a widely used benzodiazepine, (2) allopregnanolone, a naturally occurring neurosteroid and (3) XBD173, an agonist of the translocator protein (TSPO) that increases endogenous neurosteroid synthesis. Specifically, we hypothesised that α1-GABA_A_ receptor-controlled activity is attenuated by midazolam but not by allopregnanolone or XBD173. Furthermore, we suspected that LFA should be more sensitive to neurosteroids than to midazolam. Our major finding is that midazolam, on the one hand, and allopregnanolone and XBD173, on the other hand, shape ongoing activity in cortical networks in substantially different ways. As expected, the effect of midazolam was prominent during HFA and largely mediated by α1-GABA_A_ receptors. In contrast, allopregnanolone and XBD173 strongly downregulated LFA, with no effect on HFA, suggesting that these agents are not very effective in modulating α1-receptor-dependent GABAergic inhibition.

## 2. Results

### 2.1. Midazolam Shapes Cortical Up-States via α1-GABA_A_ Receptors

In cortical tissue slices, spontaneous activity is characterised by rhythmic changes between periods of sustained firing (up-states) and periods of low activity (down-states) that occur at frequencies between 0.01 and 0.5 Hertz. In the first set of experiments, we investigated whether midazolam reduces action potential firing during up-states and whether this potential effect involves α1-GABA_A_ receptors. Balk and coworkers previously demonstrated the effectiveness of midazolam in a concentration range from 0.5 nM to 100 µM [12]. Here, we used mice carrying knock-in mutations in the α2-, α3- and α5-subunits of the GABA_A_ receptor to uncover the actions of midazolam mediated exclusively via α1-GABA_A_ receptors. Mutations of a histidine residue into an arginine residue dramatically reduce midazolam binding without attenuating receptor activation by γ-aminobutyric acid [13]. Thus, in the absence of benzodiazepines, these mutations are silent, whereas receptors incorporating the mutated α-subunits are resistant to benzodiazepines. With these triple-knock-in animals (α2/3/5-knock-in mice), it was possible to isolate the effects of the drug that were mediated exclusively by α1-GABA_A_ receptors [14].

**Figure 1:**
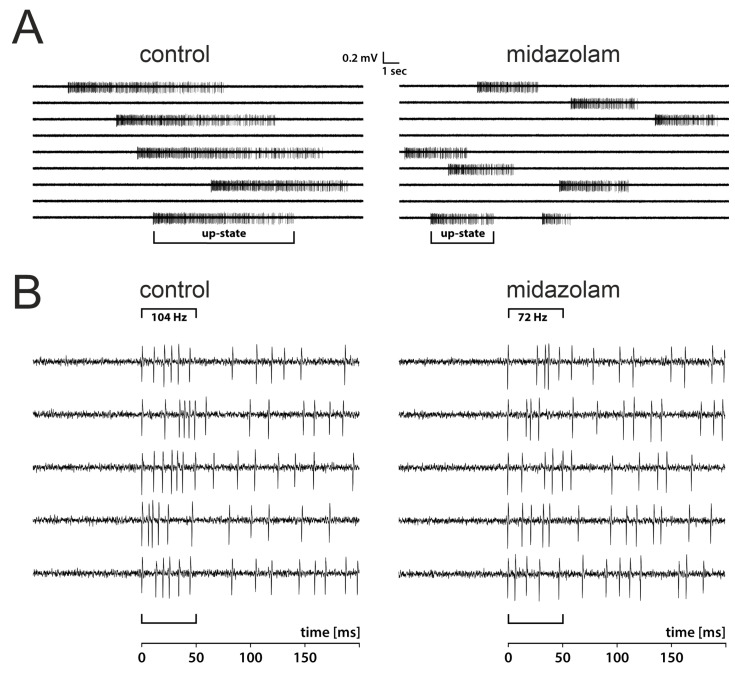
Extracellular multi-unit recordings in a cultured neocortical slice from an α_2/3/5_-knock-in mouse. We chose this experiment as an example because in this recording, the number of active units was small, and the drug effect is evident by visual inspection. (**A**) Activity patterns under control conditions (left) and in the presence of midazolam (concentration: 100 nM, right). At a low temporal resolution, active phases (up-states) can clearly be distinguished from silent phases (down-states). (**B**) The beginning of five successive up-states is shown at a higher temporal resolution. Firing rates are especially high at the onset of up-states, but then rapidly decline. Midazolam attenuated high-frequency action potential firing at the beginning of up-states by about 30% (from 104 Hz to 72 Hz).

**Figure 2:**
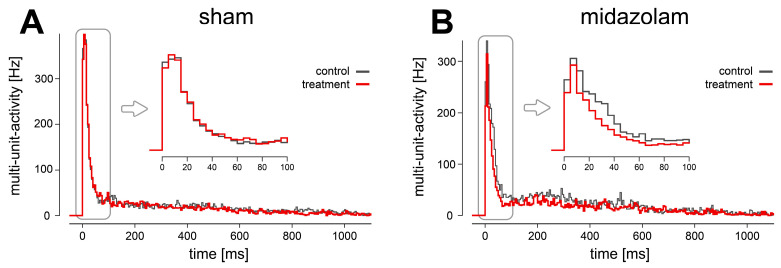
Effect of 100 nM midazolam on cortical up-states in slices from α_2/3/5_-knock-in mice. In this preparation, the drug acts only via α_1_-GABA_A_ receptors. To quantify the effects of midazolam, we extracted all up-states that were recorded in a slice under drug-free (control) or drug/sham (treatment) conditions. We subdivided the time course of the detected up-states into segments of five milliseconds each and counted the number of action potentials during these intervals. This procedure provided histograms for all detected up-states, which were then averaged to obtain the median action potential activity within up-states. (**A**) Multi-unit activity during up-states averaged across all recordings under control (black) and under sham conditions (red). The inset displays the first 100 milliseconds at a higher temporal resolution. It is evident that sham treatment did not alter the recorded multi-unit-activity. In the absence and presence of 100 nM midazolam ((**B**), red), two visibly distinct forms of activity are apparent: A short phase of high-frequency action potential activity at the beginning of the up-states, peaking around 5–20 milliseconds after its onset, and a much longer period of low-frequency action potential firing. Midazolam reduces multi-unit activity during this phase. Additionally, midazolam also has an inhibitory effect during the late phase.

Figure 1 presents data from a representative experiment in a cortical tissue slice derived from an α2/3/5-knock-in mouse. The traces display extracellular recordings of spontaneous multi-unit action potential activity under drug-free conditions (left) and after the application of 100 nM midazolam (right), a concentration that is assumed to cause deep sedation in patients [15]. Midazolam shortened the duration of up-states (Figure 1A) and reduced high-frequency-firing at the onset of up-states by about 30% (Figure 1B). The time courses shown in Figure 2 provide the median activities during up-states of all recordings. The right diagram (Figure 2B) displays the multi-unit action potential activities for the midazolam group in the absence (black line) and presence of the drug (red line).

These histograms show a clear distinction between an early phase of high-frequency activity, followed by a late phase which is characterised by sustained action potential firing at much lower discharge rates, persisting until the end of the up-states. It is apparent that midazolam reduced the initial activity-peak (Figure 2B, inset), an effect that was clearly absent in the sham group (Figure 2A).

**Figure 3:**
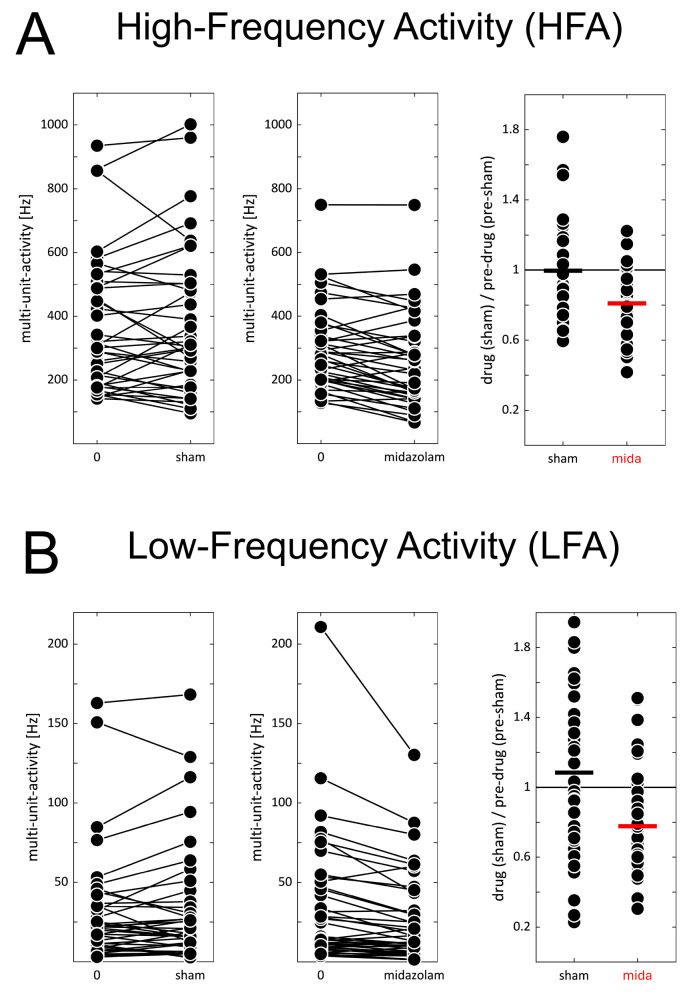
HFA and LFA before and after midazolam (100 nM) or sham treatment. (**A**) The discharge rates during the first 50 ms after the onset of up-states were determined for every single slice before (mean activity (±SD) = 182.0 ± 105.4 Hz) and during (184.6 ± 114.9 Hz) sham treatment ((**A**), left diagram) as well as in the absence (147.7 ± 66.2 Hz) and presence (127.8 ± 72.8 Hz) of midazolam ((**A**), middle). The interconnected points represent consecutive measurements in the same slice (paired samples). Dividing the action potential activity after drug administration or sham treatment by the action potential activity before the treatment yields the normalised activity. Normalised drug effects are shown in the diagram on the right. The horizontal bars represent the medians (midazolam (red bar): 0.81; CI = 0.77/0.90; sham: 1.00; CI = 0.93/1.12; two-sample t-test: *p* < 0.001). (**B**) Effects of midazolam on LFA. Unlike the sham treatment (before treatment: 46.7 ± 77.0 Hz; during treatment: 48.5 ± 79.1), midazolam (before treatment: 32.4 ± 37.5 Hz; during treatment: 24.8 ± 26.3) significantly depressed activity during LFA (midazolam (red bar): 0.78; CI = 0.72/0.90; sham: 1.08; CI = 0.97/1.26; two-sample t-test: *p* < 0.001).

Next, the effects of midazolam on high-frequency-action (HFA) and low-frequency-action (LFA) potential activity were separately analysed. For this purpose, the multi-unit firing rate was determined during the first 50 milliseconds of the up-states (= HFA) and during the time window from 75 to 1200 milliseconds after the start of the up-states (= LFA). Figure 3A displays the effect of 100 nM midazolam on HFA and the associated sham treatment. The drug significantly reduced HFA compared to the sham group (midazolam: 19% inhibition, n = 39; sham: 0% inhibition, n = 38; two-sample t-test: *p* < 0.001). In Figure 3B, the effect of 100 nM midazolam on LFA is displayed, also showing a significant decrease in action potential activity (midazolam: 22% inhibition, n = 39; sham: 8% activation, n = 38; two-sample t-test: *p* < 0.001). In conclusion, midazolam, at a concentration of 100 nM, significantly reduced both HFA and LFA by enhancing the function of α_1_-GABA_A_ receptors.

### 2.2. HFA Is More Sensitive to the Modulation of α1-GABA_A_ Receptors by Midazolam than LFA

**Figure 4:**
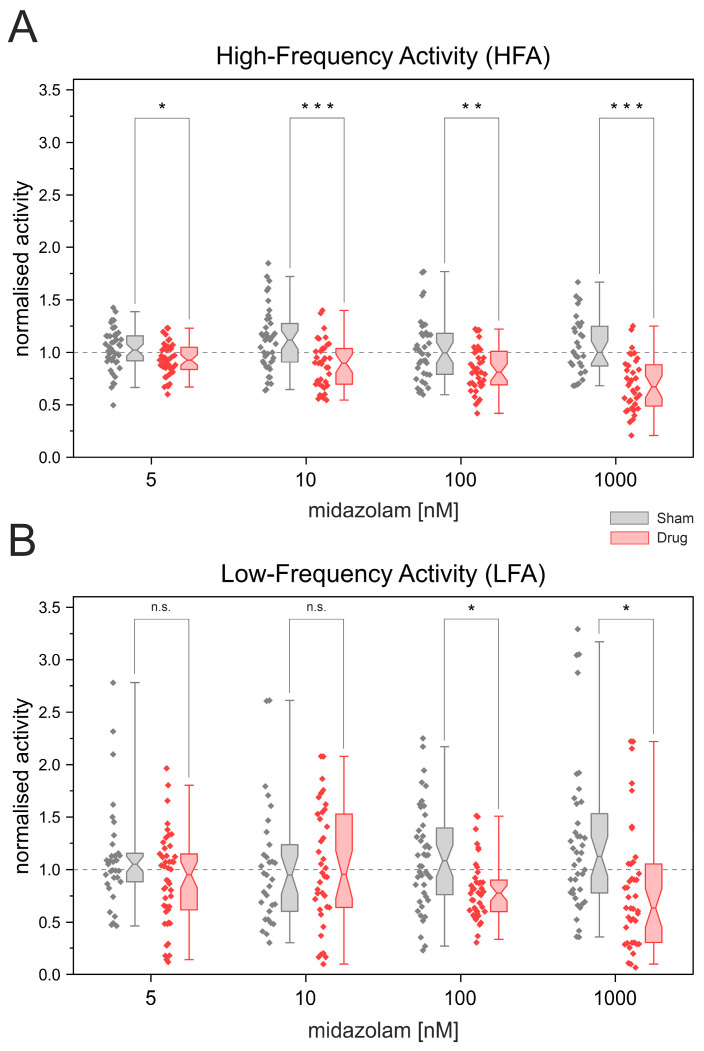
Concentration-dependent effects of midazolam on multi-unit activity during up-states in slices from α_2/3/5_-knock-in mice. Normalised activities of the drug (red) and the sham group (grey) are displayed as individual experiments (dots) and as boxplots. The middle bar in the boxplots indicates the median, the lower and upper bars represent the 25% and 75% percentiles, and the whiskers indicate the 2.5–97.5% percentiles. The notches display the 95% confidence interval of the median. A normalised activity of 1 (indicated by the dotted line) denotes that the activity is not altered by the treatment. Smaller values indicate a decrease in activity. (**A**) Effects of midazolam on HFA: Midazolam attenuated HFA in a concentration-dependent manner. The differences between the drug and sham groups were statistically significant for all tested concentrations (as indicated by the asterisks). Further details are provided in Table A1. (**B**) Effects of midazolam on LFA: Low concentrations of 5 and 10 nM midazolam did not significantly alter spontaneous action potential activity. Only higher concentrations of midazolam (100 and 1000 nM) were effective in reducing LFA (see Table A2).

Our earlier observations that diazepam [10] and zolpidem [11] suppress HFA via α1-GABA_A_ receptors align with our results displayed in Figure 2 and Figure 3. However, in the present study, midazolam dampened not only HFA, but also LFA. This raises the question of whether these two effects occur at the same or at different drug concentrations. We recently reported that 10 nM midazolam almost completely suppressed hippocampal long-term potentiation [15]. Therefore, we studied the drug’s effects on HFA and LFA at concentrations ranging from 5 to 1000 nM, which in humans are assumed to produce mild sedation and amnesia at the lowest [16] and unconsciousness at the uppermost concentration [17,18]. Experiments were conducted in tissue slices derived from α2/3/5-knock-in-mice, in which midazolam acts exclusively via α1-GABA_A_ receptors. The slices were randomly assigned either to the midazolam (red) or to the sham group (grey). The results of the individual experiments are shown in Figure 4, displayed as normalised activities, and the corresponding statistical parameters are listed in Table A1 and Table A2. Midazolam significantly reduced HFA at all tested concentrations (Figure 4A and Table A1). Concentration-dependent effects ranged from about 9% inhibition at 5 nM to 33% inhibition at 1000 nM. The actions of midazolam on LFA are provided in Figure 4B and Table A2. A significant inhibitory effect was only observed when the slices were exposed to 100 (30% inhibition, *p* < 0.001, n = 40/44) and 1000 nM (50% inhibition, *p* < 0.001, n = 41/40) midazolam. In summary, HFA appears to be more sensitive to midazolam-induced positive modulation of α1-GABA_A_ receptors than LFA.

### 2.3. HFA, but Not LFA, Is Insensitive to Allopregnanolone

In the first part of this study, we used α2/3/5-knock-in-mice in combination with the benzodiazepine midazolam to confirm our previous finding that HFA is tightly controlled by α1-GABA_A_ receptors [10,11,12]. Similar to benzodiazepines, neuroactive steroids act as positive allosteric modulators at multiple GABA_A_ receptor subtypes. In the second part of this study, we investigate whether α1-GABA_A_ receptors are among the preferred molecular targets of neurosteroids by utilising HFA as a biomarker for compounds that modulate this GABA_A_ receptor subtype. Thus, we characterised the effects of allopregnanolone, a neurosteroid and potent GABA_A_ receptor modulator, and the effects of XBD173, a substance that enhances neurosteroid de novo synthesis.

**Figure 5:**
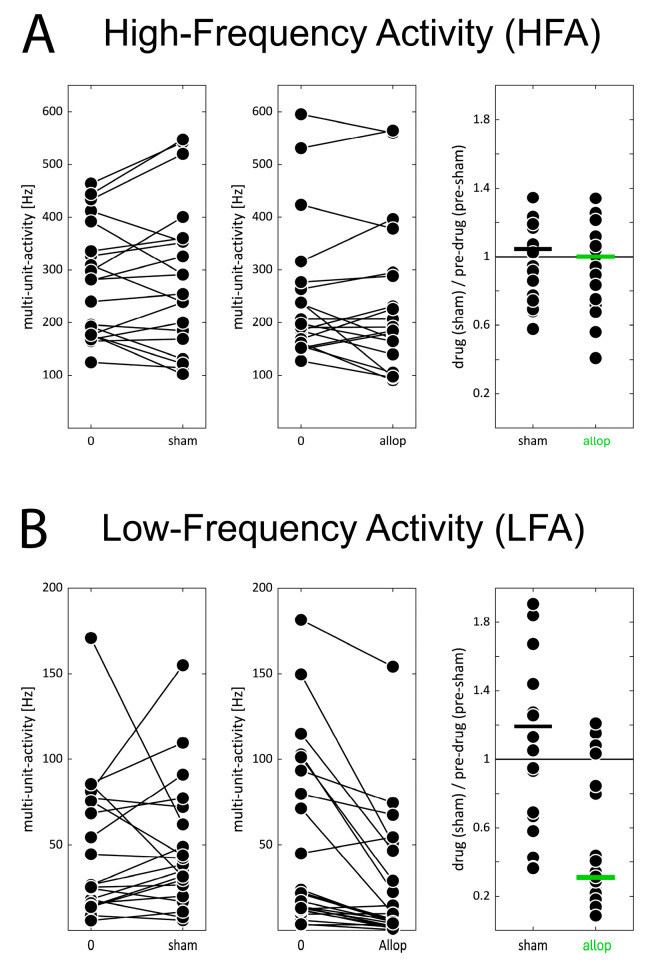
Effect of 50 nM allopregnanolone on cortical up-states in slices from wild-type mice. A detailed explanation of the symbols is provided in the legend of Figure 2. (**A**) Allopregnanolone did not significantly attenuate HFA (before sham treatment: 139.8 ± 53.7 Hz; during sham treatment: 143.5 ± 69.7 Hz; before allopregnanolone: 125.2 ± 65.5 Hz; during allopregnanolone: 120.2 ± 71.6 Hz). The medians of the normalised activities in the allopregnanolone (green horizontal bar) and the sham group were both close to 1, indicating that treatments were not effective. (**B**) In contrast to HFA, allopregnanolone was highly effective in depressing LFA (before sham treatment: 44.0 ± 38.0 Hz; during sham treatment: 44.6 ± 35.1 Hz; before allopregnanolone: 44.6 ± 47.8 Hz; during allopregnanolone: 22.7 ± 33.4 Hz).

Of particular interest here is whether these agents can affect HFA at concentrations that induce a significant decrease in overall neuronal activity. If this is not the case, α1-GABA_A_ receptors should not be regarded as one of their preferred molecular targets. First, we analysed the actions of allopregnanolone on up-states in cortical tissue slices derived from wild-type mice. The experimental design of these studies was similar to that of the previously described experiments with midazolam. Therefore, the results for the effect of allopregnanolone in Figure 5 can be compared directly to the effects of midazolam shown in Figure 3. It is evident that, for HFA, the normalised activity in both the allopregnanolone and sham groups is close to 1 (Figure 5A), indicating that allopregnanolone does not modify HFA. In contrast, LFA is strongly suppressed by allopregnanolone (Figure 5B). In other words, allopregnanolone does not exert the inhibitory effects on HFA that midazolam exerts via α1-GABA_A_ receptors, whereas LFA is markedly more sensitive to allopregnanolone than to midazolam.

### 2.4. Enhancing Neurosteroidogenesis via XBD173 Dampens LFA but Not HFA

**Figure 6:**
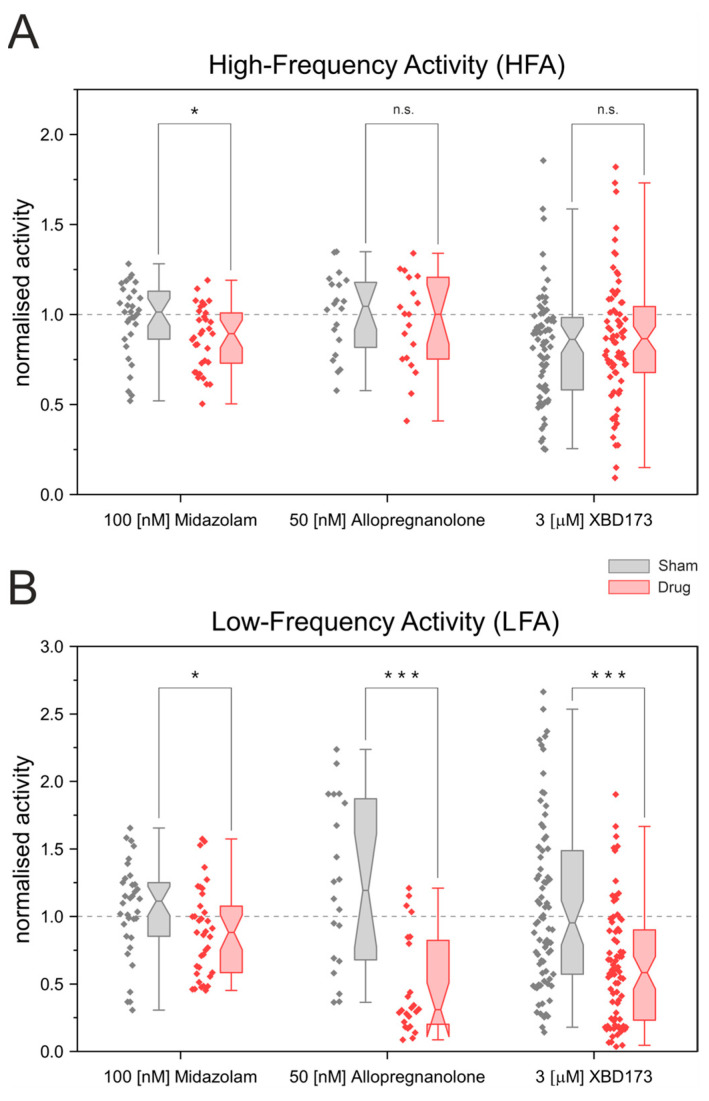
Effects of 100 nM midazolam, 50 nM allopregnanolone, and 3 µM XBD173 (red), as well as the respective sham treatments (grey) on HFA (**A**) and LFA (**B**) during cortical up-states in slices prepared from wild-type mice. The effects of the treatments are expressed as normalised activities. A detailed explanation of the symbols is provided in Figure 4.

XBD173, also known as emapunil, is a selective agonist of the translocator protein 18 kDa (TSPO) located on the outer mitochondrial membrane, particularly in glial cells [19]. It is an experimental anxiety-reducing drug without typical benzodiazepine side effects that acts by enhancing neurosteroidogenesis [20]. In the XBD173 experiments, the exposure time was increased from 12 to 30 min before action potential activity was recorded to allow enough time for the endogenous neurosteroidogenesis to occur in the tissue slices. Figure 6 and Table A3 and Table A4 compare the effects of XBD173 on HFA and LFA to those of midazolam and allopregnanolone. All recordings displayed in this figure were performed in tissue sections from wild-type mice. Similar to allopregnanolone but different from midazolam, XBD173 did not significantly alter HFA (Figure 6A). The differences in normalised activities within the sham groups (midazolam: 1.01, allopregnanolone: 1.05, XBD173: 0.86) are likely related to the longer exposure time during the XBD173 treatment. Unlike HFA, LFA was strongly suppressed by both XBD173 and allopregnanolone (Figure 6B). Thus, XBD173 and allopregnanolone showed a very similar action profile, as both lacked any notable effect on HFA but significantly reduced LFA. In contrast to XBD173 and allopregnanolone, midazolam significantly inhibited HFA. It is interesting to note that midazolam inhibited HFA and LFA to a similar extent in tissue sections from wild-type and α2/3/5-knock-in mice, supporting the hypothesis that α_1_-GABA_A_ receptors are a primary molecular target of this drug.

## 3. Discussion

### 3.1. In Vitro Studies on the Effects of Benzodiazepines on HFA Mediated by α1-GABA_A_ Receptors

**Figure 7:**
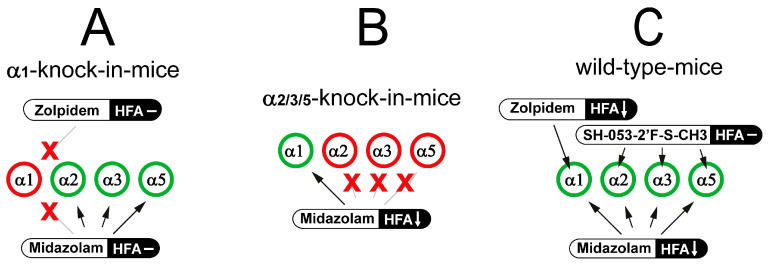
Mouse models and pharmacological tools utilised for evaluating the role of α1-GABA_A_ receptors in shaping high-frequency network activity (HFA). (**A**) Midazolam, a classical benzodiazepine, binds to the α1-, α2-, α3- and α5-subtypes of the GABA_A_ receptor. Zolpidem is a hypnotic drug that preferably binds to the benzodiazepine binding site of α1-GABA_A_ receptors. In α1-knock-in mice, a point mutation in the α1-subunit reduces binding of midazolam and zolpidem to α1-GABA_A_ receptors. As a consequence, and unlike in wild-type mice, these agents do not attenuate HFA (indicated by the white horizontal line). (**B**) In α2/3/5-knock-in-mice, α2-, α3- and α5- GABA_A_ receptor subunits are point-mutated and therefore resistant to modulation by benzodiazepines. In this mouse model, midazolam reduces HFA (downward arrow) via positive allosteric modulation of α1-GABA_A_ receptors. (**C**) In wild-type animals, midazolam and zolpidem are both effective in attenuating HFA. The experimental benzodiazepine derivative SH-053-2′F-S-CH3 is effective at α2-, α3- and α5-GABA_A_ receptors but not at α1-GABA_A_ receptors. This agent fails to reduce HFA but not LFA, suggesting that α2-, α3-, and α5-GABA_A_ receptor subtypes do not play a major role in regulating HFA.

High-frequency activity in the cerebral cortex is generated during sensory processing and cognition [21]. Drugs regularly administered to attenuate cortical activity are general anaesthetics and benzodiazepines [22,23]. On the molecular level, these agents act by increasing GABA_A_-receptor-mediated synaptic and extrasynaptic inhibition [1]. In the cerebral cortex, 80% of neurons are glutamate-releasing excitatory pyramidal cells [24]. Their cell bodies are densely occupied by α1-GABA_A_ receptors, which are mainly innervated by fast-spiking parvalbumin-positive (PV-positive) GABA-releasing basket cells [25,26]. Due to their abundance and cellular localisation, it is reasonable to assume that α1-GABA_A_ receptors are important players in shaping cortical network activity. In order to test the hypothesis that HFA in cortical slices is controlled by α1-GABA_A_ receptors, we conducted studies with two complementary animal models (Figure 7). As previously mentioned, there are four different subtypes of benzodiazepine-sensitive GABA_A_ receptors: α1-, α2-, α3- and α5-GABA_A_ receptors. In the first animal model (single knock-in animals, Figure 7A), only α1-GABA_A_ receptors were resistant to benzodiazepines, due to a point mutation in the benzodiazepine binding site. In other words, only the effects of benzodiazepines mediated by α1-GABA_A_ receptors are absent in these mice, while the physiologic function of this subtype is not altered. In the second animal model, the triple-knock-in model (Figure 7B), α2-, α3- and α5-GABA_A_ receptors had histidine-to-arginine mutations in their respective binding sites to benzodiazepines and were resistant to benzodiazepines. Therefore, in these mice, we only observed effects that are mediated by α1-GABA_A_ receptors.

Another way to explore the impact of α1-GABA_A_ receptors on HFA is to study subtype-selective drugs like zolpidem, which act mainly via this specific receptor subtype. Zolpidem suppressed HFA in tissue slices of wild-type mice (Figure 7C) but not in slices from α1-knock-in animals (Figure 7A), indicating that zolpidem-sensitive α1-GABA_A_ receptors are active during HFA [11]. Furthermore, we were able to show that in tissue sections of α1-knock-in mice, neuronal activity during up-states is not altered by low concentrations (5 nM) of midazolam [12]. In tissue sections from wild-type mice, however, a significant inhibition was observed. These findings on midazolam effects in slices derived from α1-knock-in-mice align closely with our observation that in slices from α2/3/5-knock-in-mice, already a low midazolam concentration (5 nM) significantly reduced HFA (Figure 4A). In summary, all in vitro studies with midazolam and zolpidem on tissue sections from wild-type, α1- and α2/3/5-mice lead to the conclusion that the activation of α1-GABA_A_ receptors is an essential component of the modulation of cortical HFA.

### 3.2. Behavioural and Electroencephalographic Effects of Benzodiazepines Mediated by α1-GABA_A_ Receptors

Behavioural studies in α1-knock-in mice (Figure 7A) provided evidence that the sedative effects of diazepam [13] and zolpidem [27] are absent in this mouse line. In contrast, α2/3/5-mice were sedated by midazolam [14]. Further studies in α1-knock-in-mice showed that α1-GABA_A_ receptors were responsible for the addictive effect of midazolam [4]. Moreover, studies on the same mouse line indicated that α1-GABA_A_ receptors contribute to the development of tolerance to benzodiazepines [5]. This finding implies a high plasticity in the expression of α1-GABA_A_ receptors. The enormous potential of the plasticity of GABA_A_ receptor expression is demonstrated by α1-knock-out-animals that do not possess α1-GABA_A_ receptors. The absence of this major subtype of GABA_A_ receptors appears to be compensated for by an upregulated expression of α2- and α3-GABA_A_ receptors, as α1-knock-out-animals are viable and exhibit hardly any behavioural differences from wild-type animals [28,29].

What are the electroencephalographic correlates of α1-receptor-mediated benzodiazepine effects? Tobler and coworkers compared the actions of diazepam on the spontaneous electroencephalogram in wild-type and α1-knock-in mice and found no significant differences [30]. However, in this study, high frequencies in the gamma range (>25 Hz) were not investigated. Hence, in a subsequent study, we focused specifically on benzodiazepine effects on the high-frequency bands and were able to show that in α2/3/5-knock-in (Figure 7B) and in wild-type mice (Figure 7C), high-frequency field potentials and multi-unit action potentials were significantly suppressed by diazepam [10]. In summary, α1-GABA_A_ receptors mediate the sedative and addictive actions of benzodiazepines and play a role in benzodiazepine tolerance. The bioelectrical correlate of these α1-GABA_A_ receptor dependent effects is the suppression of high-frequency neuronal activity in vivo and in vitro.

### 3.3. Allopregnanolone and XBD173 Are Not Effective in Modulating HFA

**Figure 8:**
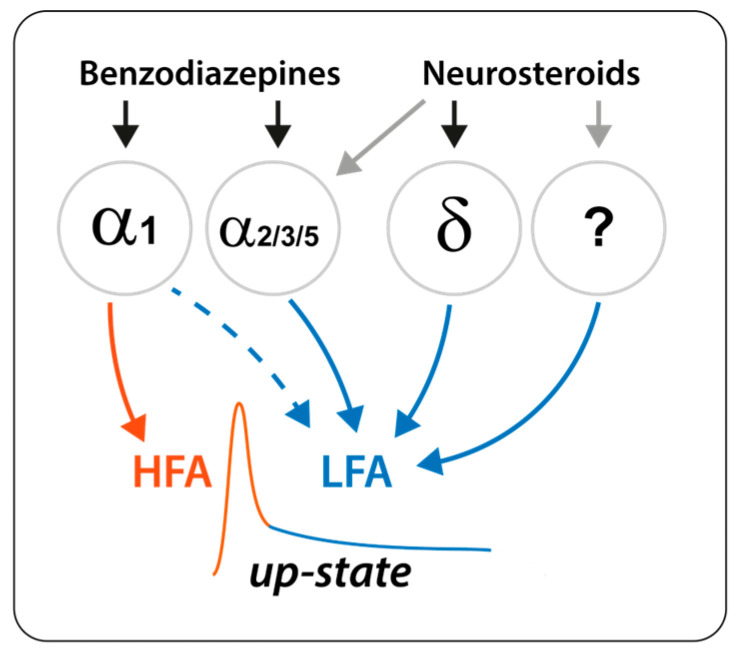
Potential molecular targets through which benzodiazepines and neurosteroids affect high- (HFA, red) and low-frequency (LFA, blue) cortical network activity. Black arrows represent proven targets, whereas grey arrows depict targets in question. More than half of all cortical GABA_A_ receptors contain an α1-subunit, making them the largest subgroup of GABA_A_ receptors in the brain. Studies using knock-in animal models have shown that HFA is attenuated by the prototypical benzodiazepine midazolam via α1-GABA_A_ receptors (red arrow). Higher concentrations of midazolam additionally inhibit LFA (dashed blue arrow). GABA_A_ receptors containing the δ-subunit are resistant to benzodiazepines but highly sensitive to neurosteroids. Neurosteroids suppress LFA while not altering HFA, suggesting that these agents do not enhance α1-receptor-mediated GABAergic inhibition. As the molecular targets of neurosteroids are less well characterised than the targets of benzodiazepines, other mechanisms may contribute to the suppression of LFA.

In sharp contrast to midazolam, allopregnanolone and XBD173 failed to alter HFA while strongly depressing LFA. How can we explain these different effect profiles, assuming that GABA_A_ receptors are also largely responsible for the actions of neurosteroids? The experimental benzodiazepine derivative SH-053-2′F-S-CH3 acts mainly via α2-, α3- and α5-GABA_A_ receptors (Figure 7C) [31,32]. Like allopregnanolone and XBD173, SH-053-2′F-S-CH3 inhibits LFA, but not HFA [11]. Thus, it appears that different classes of benzodiazepine-sensitive GABA_A_ receptor subtypes are active during either HFA or LFA (Figure 8). It remains to be clarified in further studies why neurosteroids do not alter HFA. One possible explanation is that neurosteroids do not enhance the activity of α1-GABA_A_ receptors. Yet, in current literature, there are no studies supporting this hypothesis [7]. Alternatively, it is also possible that the phosphorylation state of GABA_A_ receptors compromises neurosteroid effects mediated by α1-GABA_A_ receptors. This suggestion is based on the report that neurosteroid-induced potentiation of GABAergic synaptic currents, measured in hippocampal pyramidal cells, was suppressed by inhibitors of the cAMP-dependent protein kinase [33]. It should be noted that currently there is no direct experimental evidence to support this idea.

Finally, we have to consider the possibility that during HFA, neurosteroids inhibit interneurons releasing GABA onto α1-GABA_A_ receptors located on excitatory pyramidal cells [26,34]. Since neurosteroids are positive allosteric modulators, they are only effective at GABA-activated receptors. This idea of a presynaptic inactivation of α1-GABA_A_ receptors, although speculative, is interesting and deserves further consideration. PV-positive basket cells are a major class of GABAergic interneurons that inhibit glutamatergic pyramidal neurons via α1-GABA_A_ receptors [25,26]. They participate in shaping HFA because, unlike other cortical interneurons, they can discharge at very high rates without showing spike rate accommodation [35]. Importantly, their discharge rates are controlled by GABA_A_ receptors containing δ-subunits (δ-GABA_A_ receptors), which are highly sensitive to neurosteroids [36,37]. Thus, it seems likely that the activation of PV-positive basket cells is reduced by neurosteroids, and, as a consequence, the release of GABA onto α1-GABA_A_ receptors. It is important to note that δ-GABA_A_ receptors are not exclusively located on GABAergic PV-positive basket cells, but also on glutamatergic pyramidal cells [38]. These receptors are likely involved in the neurosteroid-induced inhibition of LFA, for which α1-GABA_A_ receptors are not required. The mechanistic implication for the contribution of extrasynaptic δ-GABA_A_ receptors in neurosteroid-induced suppression of LFA could be conceptualised as follows: During HFA, extrasynaptic δ-GABA_A_ receptors are active; however, due to the large number of active GABA-releasing synapses, the neurosteroid-enhanced modulation of δ-GABA_A_ receptors is minor compared to the GABAergic inhibition produced by synaptic receptors, such as α1-GABA_A_ receptors. Conversely, during LFA, most synaptic α1-GABA_A_ receptors are silent, firing rates of cortical neurons are low, and their membrane potential is much closer to the threshold for action potential firing. Under these conditions, neurosteroid modulation of extrasynaptic δ-GABA_A_ receptors would be substantially more effective in controlling the discharge rates of cortical neurons than during HFA.

Interestingly, there is also evidence that midazolam increases the neurosteroid synthesis of hippocampal neurons by activating TSPO [39]. Such a mechanism could hypothetically explain the inhibition of LFA induced by 100 nM midazolam in tissue sections from α2/3/5-knock-in mice (Figure 3 and Figure 4), although this appears unlikely here, as the exposure time to midazolam was considerably shorter than the exposure time in the XBD173 experiments and probably not sufficient to promote neurosteroid synthesis. Regardless of this, enhanced neurosteroid synthesis induced by midazolam would not be expected to alter HFA, as neither allopregnanolone nor XBD173 modify HFA (Figure 5 and Figure 6A).

The conclusion that GABAergic inhibition via α1-GABA_A_ receptors is not the central mechanism by which neurosteroids act is also consistent with behavioural studies. Rupprecht and coworkers reported that XBD173 counteracts panic attacks without inducing sedation and the development of tolerance [20]. This result is in line with the findings that α1-GABA_A_ receptors are involved in the sedative and tolerance-promoting properties of benzodiazepines and that neurosteroids exert their effects mainly via non-α1-GABA_A_ receptors. In summary, the results of the present study suggest that neurosteroids, unlike benzodiazepines, do not suppress HFA, a type of cortical network activity controlled by α1-GABA_A_ receptors. This feature is of particular interest for the future development of novel pharmaceuticals with improved side-effect profiles.

### 3.4. Limitations of the Present Study

This work and our previous research provide strong evidence that midazolam and neurosteroids modulate different aspects of cortical activity, specifically that neurosteroids seem to be ineffective at modulating HFA. It is important to note that our explanations discussed so far are largely based on indirect evidence, which limits the conclusions that can be drawn from this study.

#### 3.4.1. Effectiveness and Clinic Relevance of the Concentrations of the Tested Compounds

An important question concerns the concentrations of the drugs used in the present study. For midazolam, we chose a concentration of 100 nM, which, on the behavioural level, causes deep sedation. However, midazolam not only has sedative and hypnotic effects but also causes amnesia and addiction. The amnesic effect of midazolam in humans occurs at plasma concentrations ranging from 6.5 ng/mL (mild amnesia) to 109 ng/mL (profound amnesia) [16,40]. Sedative effects have been demonstrated at plasma concentrations of 56 nM and higher [40]. For deep sedation and unconsciousness in a clinical context, a blood concentration of approximately 300 ng/mL is required, corresponding to a brain concentration of around 100 nM [17,18]. We selected this concentration here because it is relevant to the clinical use of midazolam, for example, in outpatient surgery or diagnostic procedures. Midazolam is rarely used exclusively for its amnestic effects. Less is known about the concentrations at which midazolam may induce dependence. In animal studies, mice were offered sugar solutions containing midazolam at a concentration of 5 ng/mL [4]. However, the average amount consumed by the mice and the resulting blood concentrations remain unknown. Schlappi reported the lethal dose of midazolam for intravenous administration as 50,000 ng/mL [41]. Thus, midazolam has a very high therapeutic index and a wide safety margin.

For allopregnanolone, concentrations causing clinical effects such as sedation and anxiolysis are unknown. However, in a previous study, we quantified the effects of allopregnanolone in neocortical slice cultures using the GABAergic anaesthetic propofol as a reference drug [42]. At a concentration of 100 nM, allopregnanolone increased the decay time of GABA_A_-receptor-mediated synaptic currents by approximately 50%. A similar effect was observed with 250 nM propofol, a concentration that induces sedation in humans [43]. Notably, 100 nM allopregnanolone not only prolonged the decay time of GABA_A_-receptor-mediated synaptic currents but also induced a tonic current, presumably mediated by GABA_A_ receptors containing δ-subunits. In contrast, propofol did not alter GABAergic tonic currents. Accordingly, 50 nM allopregnanolone and 250 nM propofol appear to be similarly effective at the receptor level, suggesting that both drugs are capable of producing clinically relevant effects at these concentrations.

Rupprecht and colleagues evaluated the clinical efficacy of XBD173 in humans and rodents and assessed its effect on GABA_A_-receptor-mediated synaptic currents in hippocampal slices using intracellular recordings [20]. Given the limited stability of intracellular recordings, they optimised incubation to 20 min at 5 µM XBD173, which enhanced GABA_A_-receptor-mediated inhibition by ~50%. In the present study, we employed extracellular recordings, for which recording stability is less time-constrained. Accordingly, slices were incubated for 30 min at 3 µM XBD173, yielding a comparable ~50% enhancement of GABAergic inhibition. This effect is assumed to be broadly equivalent to that of 50 nM allopregnanolone.

The different exposure paradigms between midazolam and XBD173/allopregnanolone experiments could be regarded as a hindrance to the direct comparability of the compounds; accordingly, the comparison between midazolam and XBD/allopregnanolone may imply that longer exposure times (30 min) do not alter the effects produced by midazolam. However, Tokuda and colleagues demonstrated that exposing hippocampal slices for 30 min to 100 nM midazolam (10 nM was also tested but was ineffective) downregulated long-term potentiation, potentially by enhancing neurosteroid de novo synthesis [39]. To avoid this complication, we opted for a shorter exposure time to midazolam. Given that (1) allopregnanolone and XBD173 strongly attenuated LFA without affecting HFA, and (2) midazolam’s effect on HFA is abolished by point-mutating α1-GABA_A_ receptors, it seems highly unlikely that midazolam-induced neurosteroid synthesis contributes to the drug’s effect on HFA.

#### 3.4.2. HFA as a Surrogate Biomarker and Interpretation of a Lack of Effect on HFA

While the rationale for using HFA as a surrogate biomarker for α1-GABA_A_-receptor-mediated activity is clearly outlined and supported by previous work, cortical network oscillations are influenced by multiple neuronal populations and receptor systems, and we need to consider the following potential limitations to this approach: First, positive modulation of α1-GABA_A_ receptors may be obscured by negative modulatory effects mediated through other molecular targets that participate in neurosteroid signalling. Second, the hypothesis that α1-GABA_A_ receptors are insensitive to neurosteroids at the concentrations tested cannot be experimentally challenged using antagonistic drugs with specific action on these receptors. Third, the mechanistic basis underlying neurosteroid-induced inhibition of LFA remains elusive. In this regard, future studies that can demonstrate that neurosteroid-mediated inhibition of LFA is independent of α1-GABA_A_ receptors would be highly valuable and help to clarify the underlying mechanisms.

Likewise, the interpretation of the lack of HFA inhibition by allopregnanolone and XBD173 also warrants further discussion. The absence of an effect could theoretically arise from (1) the absence of α1 modulation, (2) insufficient concentration or exposure, (3) differential network penetration or (4) compensatory effects on interneurons. Both the absence of α1-GABA_A_ receptor modulation (1) and compensatory effects on interneurons (4) are plausible explanations for neurosteroids suppressing LFA, but not HFA. A differential network penetration (3) seems to be less likely since cultured brain slices are typically only a few cell layers thick, allowing compounds to rapidly dissolve into the tissue and reach their binding sites.

#### 3.4.3. Potential Influence of Strain-Related Variability Between Different Genetic Backgrounds

In the present study, we used different wild-type backgrounds, which may raise questions about the rationale and potential strain-related variability in cortical network activity or GABAergic signalling. In the first part of our manuscript, we present results that support the conclusion drawn from our previous studies—that HFA is under the control of α1-GABA_A_ receptors. The first evidence that HFA is shaped by α1-GABA_A_ receptors was provided by Balk and colleagues [12]. In this study, midazolam (5 nM) depressed HFA in cultured slices derived from C57BL/6J wild-type mice. Moreover, in slices prepared from C57BL/6J mice homozygous for a histidine-to-arginine point mutation at position 101 of the α1-GABA_A_ receptors α1 subunit (α1-knock-in mice, Figure 7A), this effect was absent. The second paper addressing the same topic employed a combination of pharmacological tools and α1-knock-in mice on a C57BL/6J background to further test the hypothesis that HFA is mitigated by drugs acting as positive modulators of α1-GABA_A_ receptors [11]. Specifically, we found that zolpidem, a nonbenzodiazepine hypnotic that preferentially binds to α1-GABA_A_ receptors via the benzodiazepine binding site, attenuated HFA in C57BL/6J wild-type mice when applied at concentrations between 100 and 2000 nM. In contrast, zolpidem was completely ineffective in attenuating HFA in slices derived from α1-knock-in mice (C57BL/6J background, Figure 7A). These studies motivated the use of HFA as a surrogate biomarker for α1-GABA_A_ receptor activity to explore whether this type of network activity is sensitive to neurosteroids. To provide further experimental evidence supporting the conclusion that HFA is controlled by α1-GABA_A_ receptors, we opted for a triple mutant (α2/3/5-knock-in mice, Figure 7B), which, however, has a different genetic background (129X1/SvJ) than the animal models used in our previous studies. Nonetheless, the results obtained with this animal model clearly support our previous findings on α1-knock-in mice (C57BL/6J background). Even if network activity differs between C57BL/6J and 129X1/SvJ mice, these differences appear to have little impact on the qualitative conclusions drawn from our experiments, since all studies indicate the involvement of α1-GABA_A_ receptors in shaping HFA.

Given the methodological limitations addressed above, discussing the translational implications of the present work remains challenging and speculative. However, if future studies confirm that α1-GABA_A_ receptors are only weakly modulated by neurosteroids, undesirable adverse effects such as tolerance and dependence may prove to be less problematic with neurosteroid-based therapies than with benzodiazepines.

## 4. Materials and Methods

### 4.1. Animals

All procedures were approved by the animal care committee (Eberhard Karls University, Tübingen, Germany) and were conducted following the institutional and federal guidelines of the German Animal Welfare Act (TierSchG). Considerable efforts were made to minimise distress and reduce the number of animals used.

For our experiments, we used organotypic tissue cultures of wild-type mice (C57BL/6J and 129X1/SvJ) and α_2/3/5_ knock-in mutants on the 129X1/SvJ background. These mutants carry histidine-to-arginine (H-to-R) point mutations (H101R, H126R, H105R) in the benzodiazepine binding site of GABA_A_ receptors containing α2, α3, and α5 subunits, respectively. This mutation does not affect the binding of the natural ligand GABA (thereby maintaining the physiological function of the receptor) but makes the receptor insensitive to modulators acting via the benzodiazepine binding site. Benzodiazepines like midazolam act as positive allosteric modulators on synaptic GABA_A_ receptors whose benzodiazepine binding site contains either an α1, α2, α3, or α5 subunit [14]. Therefore, in this knock-in mutant, benzodiazepines mediate their effects exclusively via α1-GABA_A_ receptors. The wild-type mice (C57BL/6J and 129X1/SvJ) were obtained from Charles River and the knock-in mutants (129X1/SvJ background, constituent mutant alleles: Gabra2^tm1.1Uru^, Gabra3^tm1.1Uru^, and Gabra5^tm1.1Uru^) were obtained from H.-U. Zeilhofer’s group at the University of Zurich and the ETH Zurich (Zurich, Switzerland).

### 4.2. Preparation of Organotypic Slice Cultures

Neocortical slice cultures were prepared from 2- to 5-day-old mice of both sexes as previously described [15]. In short, the mice were deeply anaesthetised with isoflurane and then decapitated. The tissue preparation was performed under aseptic conditions under a horizontal laminar flow clean bench using sterile solutions and tools. The brain was removed from the skull, and the cerebellum was removed; then the cerebrum was cut into tissue slices of 300 µm thickness. Next, each slice was manually cut into two or three separate slices and fixed onto a glass coverslip using a coagulant of chicken plasma and thrombin. The slices were then transferred into plastic tubes containing 750 µL of a nutrient medium and were maintained using the roller tube technique by Gähwiler [44]. The nutrient medium was exchanged twice a week. In order to reduce glial proliferation, antimitotics (10 μM 5-fluoro-2-deoxyuridine, 10 μM cytosine-1β-d-arabinofuranoside, 10 μM uridine) and neuronal growth factor (10 nM) were added after one day of cultivation.

### 4.3. Preparation and Application of Drugs

XBD173 (Sigma-Aldrich, Taufkirchen, Germany) was dissolved in 99.9% ethanol (Merck, Darmstadt, Germany), yielding a 10 mM (XBD173) stock solution. Allopregnanolone was dissolved in dimethyl sulfoxide (DMSO), producing a 1 mM stock solution. Midazolam (hameln pharma, Hameln, Germany) was available as a 5 mg/mL ready-to-use solution. For the experiments, solutions with the desired drug concentration were freshly prepared by dissolving the corresponding volume of stock solution in artificial cerebrospinal fluid (ACSF), consisting of 120 mM NaCl, 3.5 mM KCl, 1.13 mM NaH_2_PO_4_, 1 mM MgCl_2_, 26 mM NaHCO_3_, 1.2 mM CaCl_2_, and 11 mM D-glucose. The test solutions were applied via perfusion at a flow rate of approximately 1 mL/min, using two IPC peristaltic pumps, allowing for a replacement of at least 95% of the liquid volume in the recording chamber within 2 min [45]. Recordings were taken 12 min after the beginning of the application of the test substance in order to ensure steady-state conditions, with the exception of XDB173 and allopregnanolone, which were both applied for 30 min before the recording. This long wash-in phase was necessary to provide enough time for the endogenous neurosteroidogenesis, which is supposedly promoted by XBD173, to take place in the slice cultures. A maximum of two different drug concentrations was applied to each individual slice.

### 4.4. Extracellular Multi-Unit Recordings

For extracellular multi-unit recordings, the slice cultures were placed in a recording chamber under a microscope and were perfused with ACSF at a flow rate of 1 mL/min. The ACSF was continuously bubbled with 95% oxygen and 5% carbon dioxide to keep a stable pH of 7.4. ACSF-filled glass electrodes with a tip resistance of 3 to 5 MΩ were advanced into the tissue with micromanipulators under the optical control via microscope until stable extracellular multi-unit spike activity above 100 µV in amplitude could be detected. All experiments were conducted at 34 °C. Data were acquired using a MultiClamp 700B amplifier, the Digidata 1440 interface and Axoscope 10.7 software (Molecular Devices, San Jose, CA 95134, USA).

### 4.5. Data Processing and Statistical Analysis

The extracellular recordings were analysed using self-written scripts in MATLAB 2018b (The MathWorks Inc., Natick, MA, USA). First, the raw signals were high-pass-filtered (>300 Hz) to separate action potential activity from local field potentials. Experiments were manually excluded from further analysis if interfering electric signals were present or if the base signal or the local field potentials changed fundamentally. To separate action potentials from the neuronal background noise, only spikes surpassing a manually set threshold were counted as action potentials and were included in further data analysis.

The typical action potential firing pattern observed in slice cultures from the murine neocortex is characterised by phases of spontaneous action potential firing (up-states) alternating with silent phases (down-states; see Figure 1A). Up-states were defined as initial inter-event intervals of 75 ms following at least 500 ms neuronal silence and detected using an automated burst-detection algorithm. Within cortical up-states, two visibly distinct forms of activity can be distinguished: a short phase of initially high-frequency AP firing (HFA) at the beginning of up-states, followed by a longer phase of low-frequency AP firing (LFA; see Figure 1B). For further analysis, up-states were divided into bins of 5 ms and the average number of action potentials (discharge rate) was calculated for each bin across all up-states of a recording. Due to the variable length of the up-states, this analysis was restricted to the first 1200 ms of the up-states. To illustrate the changes in neuronal activity, we plotted these averaged multi-unit activities against time, resulting in a peri-event time histogram (PETH).

For a comparison of the drug effects on different forms of cortical network activity, the average multi-unit activity was calculated during HFA (bins 10–15) and LFA (bins 20–230) for every single recording. We then calculated the normalised effect on HFA and LFA for every experiment by dividing its average activity under drug conditions by the average activity of the respective control recording (see Figure 3 and Figure 5).

Statistical comparisons of drug vs. sham effects (Figure 4 and Figure 6) are displayed as notched boxplots (box: lower end = 25th percentile and upper end = 75th percentile; whiskers: 2.5–97.5th percentile, created with Origin 2023). The effects are presented as medians and 95% confidence intervals. We applied the Lilliefors test to each dataset to test for normal distribution. For significance testing, we utilised two-sample t-tests when both datasets were normally distributed and Wilcoxon rank sum tests when at least one dataset deviated from a normal distribution. *p*-values < 0.05 were considered significant and are indicated by asterisks. For calculating effect size, Cohen’s d for paired samples was used.

## Data Availability

Dataset available on request from the authors.

## Appendix A

**Table A1:** Concentration-dependent effects of the midazolam and sham treatments on high-frequency activity (HFA) in cortical slice cultures from α_2/3/5_-knock-in mice. Conc: concentration; Mida: midazolam group; Sham: sham group; norm: median of normalised activities; effsize: effect size, with the negative sign indicating a decrease and the positive sign indicating an increase in neuronal activity; 95%CI: 95% confidence interval of the median; n: number of experiments; distr: normal = normal distribution as determined by the Lilliefors test; *p*: *p*-values derived via t-tests or Mann–Whitney U tests when at least one dataset did not meet the assumption of normal distribution, comparing the normalised activities of the midazolam and respective sham treatments.

Conc	Mida Norm Effsize	Sham Norm Effsize	Mida 95%CI	Sham 95%CI	Mida n	Sham n	Mida Distr	Sham Distr	*p*
5 nM	0.93 −0.33	1.02 +0.06	[0.88, 0.97]	[0.97, 1.08]	43	46	normal	normal	0.0110
10 nM	0.89 −0.34	1.12 +0.30	[0.81, 0.95]	[1.02, 1.20]	37	42	normal	normal	0.0002
100 nM	0.81 −0.41	1.00 +0.05	[0.77, 0.90]	[0.93, 1.12]	39	38	normal	normal	0.0024
1000 nM	0.67 −0.45	1.00 +0.05	[0.61, 0.77]	[0.90, 1.17]	36	30	normal	/	<0.0001

**Table A2:** Concentration-dependent effects of the midazolam and sham treatments on low-frequency activity (LFA) in cortical slices from α_2/3/5_-knock-in mice. For further explanations, see Table A1.

Conc	Mida Norm Effsize	Sham Norm Effsize	Mida 95%CI	Sham 95%CI	Mida n	Sham n	Mida Distr	Sham Distr	*p*
5 nM	0.95 −0.11	1.05 +0.14	[0.78, 1.03]	[0.92, 1.13]	46	33	normal	/	0.1453
10 nM	0.95 −0.21	0.95 −0.13	[0.85, 1.20]	[0.67, 1.08]	38	31	normal	/	0.8234
100 nM	0.78 −0.37	1.08 +0.14	[0.72, 0.90]	[0.97, 1.26]	40	44	/	normal	0.0009
1000 nM	0.63 −0.25	1.13 +0.08	[0.63, 0.98]	[0.90, 1.30]	41	40	normal	/	0.0007

**Table A3:** Effects of 100 nM midazolam (Mida), 50 nM allopregnanolone (AlloP) and 3 µM XBD173 (XBD) on HFA in cortical slice cultures of wild-type mice. For further explanations, see Table A1.

	Drug Norm Effsize	Sham Norm Effsize	Drug 95%CI	Sham 95%CI	Drug n	Sham n	Drug Distr	Sham Distr	*p*
Mida	0.89 −0.41	1.01 −0.08	[0.82, 0.94]	[0.95, 1.06]	32	31	normal	/	0.0360
AlloP	1.00 −0.17	1.05 +0.12	[0.83, 1.06]	[0.91, 1.10]	19	20	normal	normal	0.4705
XBD	0.87 −0.33	0.86 −0.37	[0.78, 0.94]	[0.75, 0.90]	72	71	normal	normal	0.5231

**Table A4:** Effects of 100 nM midazolam (Mida), 50 nM allopregnanolone (AlloP) and 3 µM XBD173 (XBD) on LFA in cortical slice cultures of wild-type mice. For further explanations, see Table A1.

	Drug Norm Effsize	Sham Norm Effsize	Drug 95% CI	Sham 95% CI	Drug n	Sham n	Drug Distr	Sham Distr	*p*
Mida	0.88 −0.22	1.11 −0.01	[0.78, 0.99]	[0.93, 1.17]	37	34	normal	normal	0.0432
AlloP	0.31 −0.42	1.20 +0.02	[0.22, 0.44]	[0.97, 1.51]	24	20	/	normal	<0.0001
XBD	0.58 −0.40	0.95 −0.01	[0.46, 0.68]	[0.77, 1.11]	76	78	/	/	<0.0001
